# Cost-Effectiveness of Linkage Case Management for Hospitalized People With HIV

**DOI:** 10.1001/jamanetworkopen.2025.42105

**Published:** 2025-11-05

**Authors:** Megan Willkens, Benson Issarow, Godfrey Kisigo, Philip Ayieko, Derick Deogratias, Thomas Rutachunzibwa, Severin Kabakama, Daniel Fitzgerald, Heiner Grosskurth, Elialilia Okello, Lisa R. Metsch, Saidi Kapiga, Myung Hee Lee, Robert N. Peck, Sean M. Murphy

**Affiliations:** 1 Center for Global Health, Department of Medicine, Weill Cornell Medicine, New York, New York; 2 Mwanza Intervention Trials Unit, National Institute for Medical Research, Mwanza, Tanzania; 3 Department of Infectious Disease Epidemiology, London School of Hygiene and Tropical Medicine, London, United Kingdom; 4 Ministry of Health, Mwanza, Tanzania; 5 Department of Sociomedical Sciences, Mailman School of Public Health, Columbia University, New York, New York; 6 Department of Medicine, Weill Bugando School of Medicine, Mwanza, Tanzania; 7 Department of Population Health Sciences, Weill Cornell Medicine, New York, New York

## Abstract

**Importance:**

The Daraja randomized clinical trial was a linkage case management intervention designed to improve HIV care engagement. Understanding the economic value of this type of case management intervention is essential to decision-makers in resource-constrained settings seeking to minimize HIV-related morbidity and mortality.

**Objective:**

To evaluate the cost-effectiveness of Daraja compared with enhanced standard care from the Tanzania Ministry of Health and societal perspectives.

**Design, Setting, and Participants:**

This prospective economic evaluation was conducted alongside the Daraja randomized clinical trial at 20 hospitals in Northwestern Tanzania from March 2019 to May 2023. Participants were patients hospitalized with HIV randomly assigned 1:1 to receive either the Daraja intervention or enhanced standard care and were followed up for 12 months through March 2023. Data were analyzed from May 2024 to March 2025.

**Exposure:**

The Daraja intervention group received up to 5 sessions conducted by a social worker at the hospital, in the home, and in the HIV clinic over a 3-month period. The enhanced standard care group received predischarge HIV counseling and assistance in scheduling an HIV clinic appointment.

**Main Outcomes and Measures:**

The primary outcome was the cost per disability-adjusted life-year (DALY) averted for Daraja vs standard care, assessed from Ministry of Health and societal perspectives at 3 months (intervention) and 12 months (intervention plus follow-up).

**Results:**

Among 500 participants, the mean (SD) age was 37 [12] years, and 384 participants (77%) were female. Participants in the intervention group incurred more costs from the Tanzanian Ministry of Health perspective over the 3-month intervention period ($43.4 vs $24.0; difference, $19.4 [95% CI, $9.2 to $29.6]) and over the 12-month observation period ($101.6 vs $78.2; difference, $23.4 [95% CI, $8.0 to $38.9]). From the societal perspective, Daraja incurred more costs than standard care at 3 months ($72.4 vs $47.4; difference, $25.0 [95% CI, $2.0 to $48.0]) but not 12 months ($182.7 vs $160.5; difference, $22.2 [95% CI, −$16.4 to 60.8]). DALYs were lower for Daraja participants at both the 3-month (1.2 [95% CI, 1.1 to 1.2] DALYs averted, annualized) and 12-month (1.1 [95% CI, 1.3 to 3.7] DALYs averted) time points.

**Conclusions and Relevance:**

In this economic evaluation of the Daraja intervention, Daraja was more costly than standard care from both the Tanzanian Ministry of Health and societal perspectives; however, it was associated with improvements in DALYs. These findings suggest that Daraja has a high probability of being considered cost-effective from both economic perspectives.

## Key Points

**Question:**

Is a case management intervention to improve HIV care engagement cost-effective compared with enhanced standard care from Tanzanian Ministry of Health and societal perspectives?

**Findings:**

In this economic evaluation including 500 patients, Daraja, a linkage case management intervention administered by social workers, was more costly than enhanced standard care from both stakeholder perspectives but was associated with improvements in disability-adjusted life-years. At 12 months, Daraja’s likelihood of cost-effectiveness was approximately 0.75 across a wide range of willingness-to-pay thresholds.

**Meaning:**

The Daraja intervention had a high probability of being regarded as cost-effective according to various cost-effectiveness threshold estimates.

## Teaser

This economic evaluation examines the cost-effectiveness of the Daraja case management intervention compared with enhanced standard care for hospitalized patients with HIV from the Tanzania Ministry of Health and societal perspectives.

## Introduction

In 2023, approximately 40 million people were living with HIV, and 630 000 people died from HIV-related causes, globally.^1^ The impact of the HIV epidemic is particularly large in sub-Saharan Africa, which accounts for more than 52% of all individuals living with HIV and 41% of HIV-related deaths.^2^ Consequently, the economic burden of HIV/AIDS in sub-Saharan Africa is substantial; for example, a 2019 study found that every 1% increase in HIV/AIDS prevalence during 2000 to 2015 was associated with a 0.47% decline in per-capita income growth.^3^

Even with current antiretroviral therapy (ART), people with HIV in sub-Saharan Africa are frequently hospitalized, and approximately 30% of those hospitalized die within the first year of hospital discharge.^4,5^ There are several reasons for this, but a key factor is the failure of many individuals to remain linked with HIV care services after hospital discharge.^6^ Therefore, addressing the needs of hospitalized individuals with HIV is a key to mitigate HIV-related harms in sub-Saharan Africa.^7,8^ Our team conducted a randomized trial of a linkage case management intervention (Daraja, meaning *bridge* in Kiswahili) designed to improve HIV care engagement and found significant improvements in HIV clinic retention, ART adherence, and viral load suppression.^9,10^ However, the demonstrated effectiveness of an intervention is not typically sufficient to justify widespread adoption, especially in resource-constrained regions, like sub-Saharan Africa. Maximizing access to evidence-based care requires decision-makers to simultaneously consider the relative costs and effectiveness of alternative treatment models.

The objective of this study was to evaluate the cost-effectiveness of the Daraja intervention compared with enhanced standard care. The analysis was conducted from the perspectives of the Tanzanian Ministry of Health (MOH) and society, using the primary outcome of cost per disability-adjusted life-year (DALY) averted, examined following both the 3-month intervention and 12-month follow-up periods.

## Methods

This economic evaluation was conducted as part of the Daraja trial, approved by the ethical review committees of the National Institute for Medical Research in Tanzania, Weill Cornell Medicine, and the London School of Hygiene and Tropical Medicine. The Daraja trial was registered on ClinicalTrials.gov (NCT03858998). All participants provided written informed consent. This study was conducted in accordance with the Consolidated Health Economic Evaluation Reporting Standards (CHEERS) reporting guideline. Following guidelines from Neumann et al,^11^ an impact inventory was included to characterize potential health and nonhealth intervention impacts according to stakeholder perspective, along with justification and measurement source (eTable 1 in Supplement 1).

### Trial Summary

The Daraja trial, described elsewhere,^9^ was a single-blind, multisite, individually randomized clinical trial testing the effectiveness of a linkage case management intervention for hospitalized individuals with HIV compared with enhanced standard care conducted from March 2019 to May 2023. The primary outcome was posthospital 12-month mortality, and secondary outcomes included HIV clinic attendance, ART use, and viral load suppression. The Daraja intervention included 5 approximately 45-minute sessions delivered by a social worker over 3 months. The first session took place in the hospital, the second in the participant’s home, and sessions 3 through 5 at the HIV clinic or a location of the participant’s choosing. Enhanced standard care included predischarge HIV counseling and assistance in scheduling an HIV clinic appointment. A total of 500 participants were randomized 1:1 to receive Daraja (250 participants) or enhanced standard care (250 participants). Posthospitalization outcomes were assessed at 12 months after randomization.

The Daraja intervention did not have a significant effect on 12-month mortality, the primary outcome (17.2% among Daraja participants vs 16.8% with enhanced standard care; hazard ratio [HR], 1.01 [95% CI, 0.66-1.55]) but did lead to significant reductions in time to HIV clinic linkage (HR, 1.50 [95% CI, 1.24-1.82]) and ART initiation (HR, 1.56 [95% CI, 1.28-1.89]).^9^ The intervention group also had increased ART adherence (81.1% vs 67.6%), viral load suppression (78.6% vs 67.1%), and retention in care (87.4% vs 76.3%) at 12 months.

### Economic Evaluation Overview

A prespecified prospective economic evaluation was conducted in conjunction with Daraja, from the perspectives of the MOH and society, according to established guidelines.^11,12^ Whereas economic decision-analytic models typically rely on existing inputs from multiple sources to estimate outcomes under hypothetical scenarios, prospective trial-based evaluations use participant-level data collected at prespecified time points with instruments designed to capture all relevant cost and outcome components. As such, cost and effectiveness measures are observed jointly over time, eliminating the need for a priori assumptions about their associations and supporting the use of econometric methods designed to enhance the accuracy and validity of the estimates by addressing statistical challenges, such as confounding, skewed data, attrition, sampling imbalances, and missing data.

Select baseline participant characteristics were compared across trial groups using *t* tests for continuous variables and χ^2^ tests for categorical variables, as in prior trial-based economic evaluations.^13,14^ The resource costing method was used to assign monetary values to all nonintervention resources used by participants.^11,12^ Unit costs were collected in Tanzanian shillings, then converted to US dollars, and updated to 2023 values (eTable 2 in Supplement 1). The MOH perspective included costs that would be incurred on behalf of patients receiving the Daraja intervention in a clinical setting, as well as other health care services typically covered by the Tanzanian MOH, specifically, inpatient, outpatient, and HIV clinic resources. In addition to health care resources and costs included in the MOH perspective, the societal perspective accounted for the value of other resources potentially associated with participant’s receipt of HIV care, such as traditional healer services, time spent seeking and receiving care, and changes in workplace productivity. Differences in adjusted mean total costs between groups were evaluated in conjunction with between-group differences in DALYs. The analyses were conducted for all enrolled participants using an intent-to-treat design. The unit of observation was the 3-month intervention period, in line with the trial’s primary data collection points. Incremental cost-effectiveness ratios (ICERs) were calculated for each stakeholder perspective over the 3-month intervention and 12-month observation (intervention plus follow-up) periods.

### Cost Measures

The costs associated with implementing and sustaining the Daraja intervention were estimated using microcosting analysis, which used site visits and semistructured interviews within an activity-based costing approach to identify all resources (eg, labor, supplies) required to deliver the intervention and value them using nationally representative unit costs before summing them to generate detailed estimates.^11,12^ Additional details are available from Peck et al^9^ and in eAppendix 1 in Supplement 1. Consistent with observed strategic planning models, resource needs were evaluated in the context of a 3- to 5-year time horizon.^15^ Per the prevailing economic-evaluation guidelines, the mean per-participant cost for each intervention point of contact (excluding research-specific contacts) was estimated for the sustainment phase of Daraja. The sustainment phase includes ongoing intervention costs incurred following the intervention reaching a steady state, when operations, and thus key resources, stabilize and remain consistent over time. Sustainment costs included office space, client sessions, case manager travel, and client management (eg, follow-up calls).

### Tanzanian MOH Perspective Resources and Other Resources

Participant use of nonstudy health care services was self-reported using a customized version of the Non-study Medical and Other Services (NMOS) form (eTable 3 in Supplement 1).^16,17,18,19,20,21^ Data were collected at baseline and every 3 months thereafter over the 12-month observation period. The process was overseen by a trained research assistant, either in-person or over the phone. Health care services captured via the NMOS included: hospital inpatient, outpatient, and HIV clinic visits. The reliability and validity of self-reported health care resource utilization have consistently been demonstrated for recall periods similar to those in our study.^22,23,24,25,26,27^ Additional resources and costs captured via the NMOS included traditional healer services, other out-of-pocket expenditures incurred by participants as part of their care, time spent receiving and pursuing care, and workplace productivity, measured as the income earned from time spent working.

### Effectiveness

Effectiveness was measured using DALYs averted. The DALY is a generalizable measure that is commonly used in global health cost-effectiveness analyses.^11,28^ It integrates morbidity and mortality, by summing years of life lost due to premature mortality and years lived with disability. The years of life lost component of the DALY allows it to measure intervention-associated changes in the lifetime burden of a condition, even when conducted over a shorter time horizon. Years lived with a disability is calculated by weighting an individual’s time spent in a particular health state by the disability weight associated with that state, which ranges from 0 (perfect health) to 1 (equivalent to death). Disability weights were assigned to all participants at each 3-month time point depending on their HIV disease status and any other comorbid conditions, ascertained according to medical records independently reviewed by ^2^ clinicians. A detailed explanation of how DALYs were estimated is provided in eAppendix 2 in Supplement 1.

### Cost-Effectiveness

ICERs were used to assess the cost-effectiveness of Daraja relative to enhanced standard care at both 3 months (intervention period) and 12 months (intervention + follow-up periods). ICERs were calculated as the between-group difference in adjusted mean costs, including the mean intervention sustainment cost, divided by the difference in adjusted mean DALYs. Four ICERs were calculated to account for the difference in resources and costs relevant to each stakeholder perspective, as well as the 2 timeframes being examined. ICERs were compared with 2 country-level cost-effectiveness thresholds established by Pichon et al^29^ and Woods et al.^30^

### Statistical Analysis

Multivariable generalized linear model (GLM) regressions with a gaussian distributed outcome were used to estimate all adjusted mean resource and effectiveness values at each 3-month time point, according to study group. Resources and costs were classified and analyzed according to the following categories: the Daraja intervention, inpatient and outpatient care, HIV clinic services, and other societal factors. Regarding effectiveness, adjusted mean disability weights and years of life lost were estimated via GLM prior to being used to calculate DALYs. Additional details are provided in eAppendix 3 in Supplement 1.

All multivariable regressions were subsequently run within a nonparametric bootstrapping procedure, and 95% CIs were used to assess between-group differences in the 3-month and 12-month adjusted mean values. Cost differences were tested at the category level, then summed according to stakeholder perspective and tested. Uncertainty around the ICERs was assessed by constructing cost-effectiveness acceptability curves using data from the bootstrapping analyses. Cost-effectiveness acceptability curves display the probability that an ICER will fall below a stakeholder’s willingness-to-pay (WTP) threshold per DALY averted, thus being considered cost-effective. A more in-depth discussion regarding uncertainty evaluation is provided in eAppendix 4 in Supplement 1. Missing data bias was addressed using inverse probability weighting within the GLM framework (eAppendix 5 in Supplement 1).

Sensitivity analyses were defined a priori and focused on assessing the robustness of the health care cost findings to alternative estimation techniques; specifically, the adjusted GLM results used in this study were compared with results from adjusted and unadjusted ordinary least-squares regressions. Ordinary least-squares analysis is advantageous in terms of transparency and simplicity of computation, while GLM is generally more robust and efficient when modeling health care costs, given their propensity for right-skewness and heteroscedasticity. Additionally, findings were assessed for sensitivity to missing variable bias by comparing the primary findings adjusted using inverse probability weighting to those assessed via listwise deletion.

*P* values were 2-sided, and statistical significance was set at *P* ≤ .05. All analyses were conducted using Stata version 18.0 (StataCorp). Data were analyzed from May 2024 to March 2025.

## Results

### Study Participants

Among 500 participants, the mean (SD) age was 37 (XX) years, and 384 participants (77%) were female. Baseline characteristics were similar by study group on all measures, except for age and HIV and ART status at enrollment (Table 1). Participants randomized to Daraja were older (mean [SD] age, 38 [12] years vs 36 [11] years), and more likely to be newly diagnosed and not yet receiving ART group (211 participants [84%] vs 191 participants [76%]) compared with those in the enhanced standard care group. Most participants had completed at least a primary education (331 participants [67%]), and half of participants were married or cohabiting (248 participants [50%]).

**Table 1.** Select Baseline Characteristics of Daraja Study Participants

Characteristic	Patients, No. (%)	
	Daraja (n = 250)	Standard care (n = 250)
Sex		
Female	190 (76)	194 (78)
Male	60 (24)	56 (22)
Age, mean (SD), y^a^	38 (12)	36 (11)
Married or cohabitating	131 (52)	117 (47)
Primary school completion	165 (66)	169 (68)
HIV/ART status^a^		
Newly diagnosed HIV	211 (84)	191 (76)
Discontinued ART (>7 d)	39 (16)	59 (24)

Abbreviation: ART, antiretroviral therapy.

^a^ Difference is statistically significant at α = .05.

### Cost

The estimated mean costs and outcomes for the MOH and societal perspectives at 3 and 12 months are displayed in Table 2. From the MOH perspective, participants in the intervention group incurred more costs over the 3-month intervention period ($43.4 vs $24.0; difference, $19.4 [95% CI, $9.2 to $29.6]) and over the 12-month observation period ($101.6 vs $78.2; difference, $23.4 [95% CI, $8.0 to $38.9]). The mean per-participant cost associated with the 3-month Daraja intervention itself was $16.9 (95% CI, $16.2 to $17.6). Costs associated with inpatient and outpatient services did not differ significantly between the intervention vs standard care groups at either time period (3 months: difference, −$0.3 [95% CI, −$10.2 to $9.5]; 12 months: difference, $1.5 [95% CI, −$12.1 to $15.3]). At 3 months, the cost of HIV clinic services was significantly higher in the Daraja group ($18.9 vs $16.1; difference, $2.8 [95% CI, $0.4 to $5.2) but not significantly different over the 12-month observation period ($61.1 vs $56.1; difference, $5.0 [95% CI, −$1.6 to $11.5]).

**Table 2.** Estimated Mean Costs and Outcomes of Daraja Intervention vs Standard Care

Variable	Cost, 2023 $US^a^					
	3 mo			12 mo		
	Daraja	Standard care	Difference (95% CI)	Daraja	Standard care	Difference (95% CI)
MOH perspective						
Linkage intervention	16.9	0	16.9 (16.2 to 17.6)	16.9	0	16.9 (16.2 to 17.6)
Inpatient and outpatient services	7.6	7.9	−0.3 (−10.2 to 9.5)	23.6	22.1	1.5 (−12.1 to 15.3)
HIV clinic services	18.9	16.1	2.8 (0.4 to 5.2)	61.1	56.1	5.0 (−1.6 to 11.5)
Total	43.4	24.0	19.4 (9.2 to 29.6)	101.6	78.2	23.4 (8.0 to 38.9)
Societal perspective						
Additional resources	29.0	23.4	5.6 (−10.7 to 22.0)	81.1	82.3	–1.2 (−32.8 to 30.4)
Total	72.4	47.4	25.0 (2.0 to 48.0)	182.7	160.5	22.2 (−16.4 to 60.8)
Annualized DALYs, No.	3.5	4.7	−1.2 (−1.2 to −1.1)	3.5	4.6	−1.1 (−3.7 to 1.3)

Abbreviation: DALY, disability-adjusted life-year.

^a^ All models adjusted for age, sex, marital status, HIV/antiretroviral therapy status, education, and enrollment hospital.

The costs associated with additional societal resources did not differ significantly between study groups at 3 months (difference, 5.6 [95% CI, −$10.7 to $22.0]) or 12 months (difference, –1.2 [95% CI, −$32.8 to $30.4). Adding these costs to those from the MOH perspective resulted in the 3-month total social cost differential remaining significant ($72.4 vs $47.4; difference, $25.0 [95% CI, $2.0 to $48.0]), but the 12-month societal cost difference lost significance ($182.7 vs $160.5; difference, $22.2 [95% CI, −$16.4 to $60.8]).

### Effectiveness and Cost-Effectiveness

DALYs were lower for Daraja participants at both 3 and 12 months. The 3-month DALY result was converted to an annualized value for ease of interpretation; however, the 3-month ICERs were calculated using the nonannualized value. At 3 months, Daraja was associated with a mean annualized figure of 1.2 (95% CI, 1.1 to 1.2) DALYs averted (3.5 vs 4.7 DALYs). The difference at 12 months was not statistically significant (3.5 vs 4.6 DALYs; difference, −1.1 [95% CI, −3.7 to 1.3] DALYs).

The ICERs for both the MOH and societal perspectives by time point are displayed in Table 3. The 3-month point estimate for the MOH perspective indicated a mean cost of $67 to avoid 1 DALY for the Daraja intervention compared with standard care, but by 12 months, this figure decreased to $21 per DALY averted. From a societal perspective, the mean cost of averting 1 DALY compared with standard care at 3 months was $87, decreasing to $13 per DALY averted at 12 months.

**Table 3.** Incremental Cost-Effectiveness Ratios for Daraja Intervention

Perspective	Incremental cost-effectiveness ratio^a^	
	3 mo	12 mo
Tanzanian Ministry of Health	$67	$21
Societal	$87	$13

^a^ Calculated as cost / disability-adjusted life-years averted.

### Uncertainty

Cost-effectiveness acceptability curves displaying the uncertainty surrounding each of the ICER values are presented in the Figure. From the MOH perspective, the likelihood that Daraja would be considered cost-effective at 3 months plateaued at approximately 0.3, with a WTP threshold of $20 000 per DALY averted. The likelihood of cost-effectiveness for Daraja is substantially higher during the 12-month observation period, exceeding 0.70 at a WTP value less than $100 per DALY averted. From a societal standpoint, the likelihood of cost-effectiveness for Daraja also plateaued at approximately 0.3 for the 3-month intervention period, but at WTP threshold of more than $30 000 per DALY averted. For the 12-month period, the likelihood of cost-effectiveness closely mirrored the 12-month findings for the MOH perspective, quickly surpassing a 0.70 probability before stabilizing less than 0.80.

**Figure.**
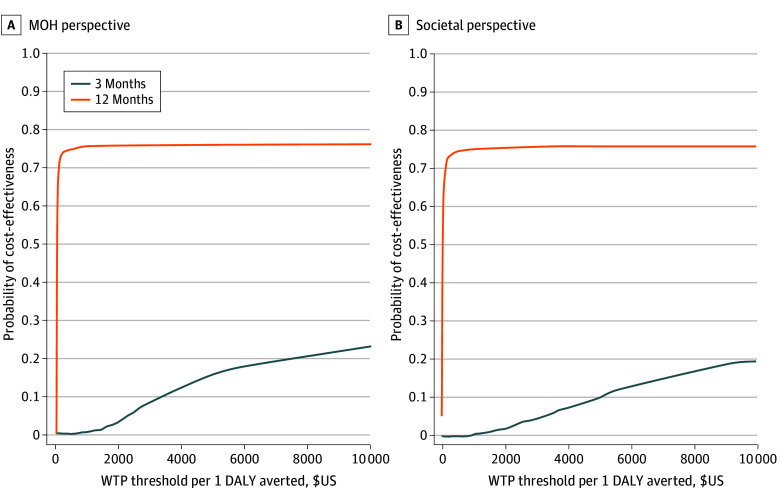
Probability of Daraja Cost Effectiveness at 3 and 12 Months by Willingness-to-Pay (WTP) Threshold DALY indicates disability-adjusted life-year; MOH, Tanzania Ministry of Health.

### Sensitivity

The results from the sensitivity analyses are displayed in eFigure 1 in Supplement 1. The analyses produced mean cost differences that ranged from $14 000 to $20 000 at 3 months, and $8000 to $28 000 at 12 months. Although the point estimates varied some across statistical models and missing data approaches, the cost differences were consistently positive, supporting the primary GLM findings.

## Discussion

To our knowledge, this is the first prospective economic evaluation to assess the cost-effectiveness of a strengths-based case management intervention for HIV in sub-Saharan Africa, adding to the limited research of the economic value of patient-based HIV treatment interventions in low- and middle-income settings.^31^ Despite other available interventions assessing posthospital outcomes across the world, 1 study in the US has been reported cost-savings associated with reduced hospital admission.^32^

Over the 3-month intervention, the mean net cost of Daraja from the MOH perspective was $19.4 per participant compared with enhanced standard care. The cost was driven by the intervention itself ($16.9), and a small increase in utilization of HIV clinic services ($2.8). By the end of the 12-month observation period, the mean cost to the MOH of Daraja had increased to $23.6. Incorporating the value of the additional resources for the societal perspective increased the mean total cost of both groups to $25.0 at 3 months and $22.2 at 12 months; the variability in social costs across participants contributed to significant differences between groups at 3 months but lost significance at 12 months.

Daraja prevented the loss of a mean of 1.2 years of full health (annualized) over the 3-month intervention period, after accounting for changes in time spent with a disability and years of life lost. At the end of the 12-month observation period, the reduction in DALYs associated with Daraja was similar, at 1.1, but this difference was no longer statistically significant. The difference observed was possibly due to better ART adherence among Daraja participants, leading to more favorable disability weights according to HIV condition. Similarly, the intervention group experienced lower rates of opportunistic infections and other HIV-related conditions; thus, this finding is reflective of a difference in morbidity, not mortality.

Although there is no standardized cost-effectiveness threshold for most low- and middle-income countries, various studies have sought to establish appropriate thresholds based on their health expenditures per capita and increasing life expectancies. Pichon-Riviere et al^29^ estimated a cost-effectiveness threshold of $251 per DALY averted ($298 in 2023 USD) for Tanzania, while Woods et al^30^ recommended a threshold range of $45 to $912 ($58-$1185 in 2023 USD). Daraja’s probability of being cost-effective at 12 months from both the MOH and societal perspectives ranged from approximately 65% at $58 to 76% at $1185, with a probability of 74% at $298 per DALY averted.^30^ Alternatively, from 2001 to 2016, the World Health Organization used thresholds based on per-capita gross domestic product (GDP) to define cost-effectiveness in low- and middle-income settings.^33^ Although no longer used officially, these thresholds still provide a useful benchmark. The World Health Organization suggested that interventions with an ICER at or below the per-capita GDP should be considered highly cost-effective. Tanzania’s 2023 GDP per capita of $1211 aligns closely with the upper range provided by Woods et al.^30^

The cost-effectiveness of other HIV treatment interventions from sub-Saharan Africa is variable depending on the scope of the intervention. According to a systematic review by Sarkar et al,^31^ the median ICER for treatment-as-prevention interventions (eg, ART for discordant couples^34^) was $1080 (in 2023 USD) per DALY averted and ICERs for early ART initiation interventions (eg, home testing and counselling^35^ and expanding ART eligibility^36^) ranged from $264 to $2004 (in 2023 USD) per DALY averted, while structural interventions for viral suppression (eg, partner notification to reduce HIV transmission^37^) were generally dominant. Most of these cost-effectiveness analyses were decision-analysis or deterministic compartmental simulation models, compared with the econometrics techniques used in this study. Additionally, most of these interventions were focused on early treatment and prevention, unlike Daraja, which sought to target patients with advanced HIV disease. Moreover, 10% of the HIV treatment interventions assessed for cost-effectiveness used decision analytic models, which provide less accuracy and internal validity compared with prospective trial-based evaluations because they rely on assumptions applied to existing epidemiologic data rather than observed participant data.

### Strengths and Limitations

This study has some strengths. In low-resource settings, it is critical to ensure that resources are put to their best use. This study helps address an important gap in the literature thanks to a prospective and comprehensive economic evaluation alongside an innovative strengths-based case management intervention for HIV in sub-Saharan Africa. Furthermore, the study deploys a novel application of DALYs via participant-level data, which allowed us to consider changes in overall well-being throughout the entire observation period using a generalizable global scale.

Despite these strengths associated with prospective trial-based economic evaluations, this study also has some limitations. The primary concerns pertain to generalizability, as a function of the trial’s eligibility criteria and resource-intensive setting. For example, due to the enhanced standard care, it is possible that participants in the control group had better outcomes than they would have otherwise, potentially diluting the intervention’s effect and, thereby, cost-effectiveness. However, where possible, steps were taken to mitigate these limitations, such as the microcost analysis focusing on the resources and costs that would be required to manage the intervention outside a clinical trial setting. Another limitation pertains to how self-reported health care and other resources were used. In particular, at each visit, participants were asked to report on their resource utilization with an anchor point of prior 30 days, as opposed to since last assessment. Thus, to estimate costs incurred by participants throughout the observation period, in instances where a participant had missed a study visit but returned for the next, the assumption was made that their resource utilization for the most recent 30 days was representative of the entire gap in data collection. An additional limitation was censored or missing data. However, missingness was minimal and was managed via inverse probability weighting, a widely accepted method to correct missing-variable bias.^38^

## Conclusions

In this prospective economic evaluation, the Daraja intervention was more costly than enhanced standard care from both the MOH and societal perspectives; however, it was associated with improvements in DALYs at 3 months. According to various cost-effectiveness thresholds, the intervention had a high probability of being considered cost-effective from the perspectives of the Tanzanian MOH and society.

## Notes

Supplement 1. **eAppendix 1.** Microcosting analysis **eAppendix 2.** Disability-Adjusted Life-Years (DALYs) **eAppendix 3.** Statistical analyses **eAppendix 4.** Uncertainty **eAppendix 5.** Missing data **eTable 1.** Impact Inventory **eTable 2.** Unit Costs (2023 USD) **eTable 3.** Non-Medical and Other Services (NMOS) form **eFigure.** Sensitivity analyses
eReferences.

Supplement 2.
Data Sharing Statement

## References

[1] World Health Organization. The Global Health Observatory: HIV. Updated 2024. Accessed February 17, 2025. https://www.who.int/data/gho/data/themes/hiv-aids

[2] UNAIDS. Eastern and Southern Africa regional profile—2024 global AIDS update. Updated 2024. Accessed February 17, 2025. https://www.unaids.org/sites/default/files/media_asset/2024-unaids-global-aids-update-eastern-southern-africa_en.pdf?t

[3] Nketiah-Amponsah E, Abubakari M, Baffour PT. Effect of HIV/AIDS on economic growth in sub-Saharan Africa: recent evidence. Int Adv Econ Res. 2019;25(4):469-480. doi:10.1007/s11294-019-09754-3

[4] Ford N, Patten G, Rangaraj A, Davies MA, Meintjes G, Ellman T. Outcomes of people living with HIV after hospital discharge: a systematic review and meta-analysis. Lancet HIV. 2022;9(3):e150-e159. doi:10.1016/S2352-3018(21)00329-5 35245507 PMC8905089

[5] Peck RN, Wang RJ, Mtui G, . Linkage to primary care and survival after hospital discharge for HIV-infected adults in Tanzania: a prospective cohort study. J Acquir Immune Defic Syndr. 2016;73(5):522-530. doi:10.1097/QAI.000000000001107 27846069 PMC5129656

[6] Okello ES, Peck RN, Issarow B, . “Ashamed of being seen in an HIV clinic”: a qualitative analysis of barriers to engaging in HIV care from the perspectives of patients and healthcare workers in the Daraja clinical trial. BMC Public Health. 2025;25(1):69. doi:10.1186/s12889-024-21231-z 39773172 PMC11706178

[7] World Health Organization. Providing care to people with advanced HIV disease who are seriously ill. Updated 2023. Accessed February 17, 2025. https://www.who.int/publications/i/item/9789240068650

[8] Burke RM, Feasey N, Rangaraj A, . Ending AIDS deaths requires improvements in clinical care for people with advanced HIV disease who are seriously ill. Lancet HIV. 2023;10(7):e482-e484. doi:10.1016/S2352-3018(23)00109-1 37301220 PMC7614731

[9] Peck RN, Issarow B, Kisigo GA, . Linkage case management and posthospitalization outcomes in people with HIV: the Daraja randomized clinical trial. JAMA. 2024;331(12):1025-1034. doi:10.1001/jama.2024.2177 38446792 PMC10918579

[10] Kisigo GA, Issarow B, Abel K, . A social worker intervention to reduce post-hospital mortality in HIV-infected adults in Tanzania (Daraja): study protocol for a randomized controlled trial. Contemp Clin Trials. 2022;113:106680. doi:10.1016/j.cct.2022.106680 35032664 PMC8882676

[11] Neumann P, Sanders G, Russell L, Siegel J, Ganiats T, eds. Cost-Effectiveness in Health and Medicine. 2nd ed. Oxford University Press; 2017.

[12] Glick H, Doshi J, Sonnad S. In: Polsky D, ed. Economic Evaluation in Clinical Trial. Oxford University Press; 2014. doi:10.1093/med/9780199685028.001.0001

[13] Murphy SM, Polsky D. Economic evaluations of opioid use disorder interventions. Pharmacoeconomics. 2016;34(9):863-887. doi:10.1007/s40273-016-0400-5 27002518 PMC5572804

[14] Onuoha EN, Leff JA, Schackman BR, McCollister KE, Polsky D, Murphy SM. Economic evaluations of pharmacologic treatment for opioid use disorder: a systematic literature review. Value Health. 2021;24(7):1068-1083. doi:10.1016/j.jval.2020.12.023 34243831 PMC8591614

[15] Steiner GA. Strategic Planning. Simon and Schuster; 2010.

[16] Polsky D, Glick HA, Yang J, Subramaniam GA, Poole SA, Woody GE. Cost-effectiveness of extended buprenorphine-naloxone treatment for opioid-dependent youth: data from a randomized trial. Addiction. 2010;105(9):1616-1624. doi:10.1111/j.1360-0443.2010.03001.x 20626379 PMC2967450

[17] Murphy SM, Campbell ANC, Ghitza UE, . Cost-effectiveness of an internet-delivered treatment for substance abuse: data from a multisite randomized controlled trial. Drug Alcohol Depend. 2016;161:119-126. doi:10.1016/j.drugalcdep.2016.01.021 26880594 PMC4792755

[18] Murphy SM, Polsky D, Lee JD, . Cost-effectiveness of extended release naltrexone to prevent relapse among criminal justice-involved individuals with a history of opioid use disorder. Addiction. 2017;112(8):1440-1450. doi:10.1111/add.13807 28239984 PMC5503784

[19] Murphy SM, McCollister KE, Leff JA, . Cost-effectiveness of buprenorphine-naloxone versus extended-release naltrexone to prevent opioid relapse. Ann Intern Med. 2019;170(2):90-98. doi:10.7326/M18-0227 30557443 PMC6581635

[20] Jalali A, Jeng PJ, Polsky D, . Cost-effectiveness of extended-release injectable naltrexone among incarcerated persons with opioid use disorder before release from prison versus after release. J Subst Abuse Treat. 2022;141:108835. doi:10.1016/j.jsat.2022.108835 35933942 PMC9508988

[21] Lu T, Ryan D, Cadet T, . Cost-effectiveness of implementation facilitation to promote emergency department-initiated buprenorphine for opioid use disorder. Ann Emerg Med. 2025;85(3):205-213. doi:10.1016/j.annemergmed.2024.10.001 39570250 PMC11845297

[22] Wallihan DB, Stump TE, Callahan CM. Accuracy of self-reported health services use and patterns of care among urban older adults. Med Care. 1999;37(7):662-670. doi:10.1097/00005650-199907000-00006 10424637

[23] Bhandari A, Wagner T. Self-reported utilization of health care services: improving measurement and accuracy. Med Care Res Rev. 2006;63(2):217-235. doi:10.1177/1077558705285298 16595412

[24] Harlow SD, Linet MS. Agreement between questionnaire data and medical records: the evidence for accuracy of recall. Am J Epidemiol. 1989;129(2):233-248. doi:10.1093/oxfordjournals.aje.a115129 2643301

[25] Roberts RO, Bergstralh EJ, Schmidt L, Jacobsen SJ. Comparison of self-reported and medical record health care utilization measures. J Clin Epidemiol. 1996;49(9):989-995. doi:10.1016/0895-4356(96)00143-6 8780606

[26] Brown JB, Adams ME. Patients as reliable reporters of medical care process. Recall of ambulatory encounter events. Med Care. 1992;30(5):400-411. doi:10.1097/00005650-199205000-00003 1583918

[27] Short ME, Goetzel RZ, Pei X, . How accurate are self-reports: analysis of self-reported health care utilization and absence when compared with administrative data. J Occup Environ Med. 2009;51(7):786-796. doi:10.1097/JOM.0b013e3181a86671 19528832 PMC2745402

[28] Charalampous P, Polinder S, Wothge J, von der Lippe E, Haagsma JA. A systematic literature review of disability weights measurement studies: evolution of methodological choices. Arch Public Health. 2022;80(1):91. doi:10.1186/s13690-022-00860-z 35331325 PMC8944058

[29] Pichon-Riviere A, Drummond M, Palacios A, Garcia-Marti S, Augustovski F. Determining the efficiency path to universal health coverage: cost-effectiveness thresholds for 174 countries based on growth in life expectancy and health expenditures. Lancet Glob Health. 2023;11(6):e833-e842. doi:10.1016/S2214-109X(23)00162-6 37202020

[30] Woods B, Revill P, Sculpher M, Claxton K. Country-level cost-effectiveness thresholds: initial estimates and the need for further research. Value Health. 2016;19(8):929-935. doi:10.1016/j.jval.2016.02.017 27987642 PMC5193154

[31] Sarkar S, Corso P, Ebrahim-Zadeh S, Kim P, Charania S, Wall K. Cost-effectiveness of HIV prevention interventions in Sub-Saharan Africa: a systematic review. EClinicalMedicine. 2019;10:10-31. doi:10.1016/j.eclinm.2019.04.006 31193863 PMC6543190

[32] Ford N, Rangaraj A, Jarvis JN, . Interventions to support people with HIV following hospital discharge: a systematic review. Open Forum Infect Dis. 2025;12(4):ofaf175. doi:10.1093/ofid/ofaf175 40212034 PMC11983270

[33] Bertram MY, Lauer JA, De Joncheere K, . Cost-effectiveness thresholds: pros and cons. Bull World Health Organ. 2016;94(12):925-930. doi:10.2471/BLT.15.164418 27994285 PMC5153921

[34] Wall KM, Inambao M, Kilembe W, . HIV testing and counselling couples together for affordable HIV prevention in Africa. Int J Epidemiol. 2019;48(1):217-227. doi:10.1093/ije/dyy203 30358840 PMC6380312

[35] Smith JA, Sharma M, Levin C, . Cost-effectiveness of community-based strategies to strengthen the continuum of HIV care in rural South Africa: a health economic modelling analysis. Lancet HIV. 2015;2(4):e159-e168. doi:10.1016/S2352-3018(15)00016-8 25844394 PMC4384819

[36] Granich R, Kahn JG, Bennett R, . Expanding ART for treatment and prevention of HIV in South Africa: estimated cost and cost-effectiveness 2011-2050. PLoS One. 2012;7(2):e30216. doi:10.1371/journal.pone.0030216 22348000 PMC3278413

[37] Rutstein SE, Brown LB, Biddle AK, . Cost-effectiveness of provider-based HIV partner notification in urban Malawi. Health Policy Plan. 2014;29(1):115-126. doi:10.1093/heapol/czs140 23325584 PMC3872371

[38] Seaman SR, White IR. Review of inverse probability weighting for dealing with missing data. Stat Methods Med Res. 2013;22(3):278-295. doi:10.1177/0962280210395740 21220355

