# Supplementary material for: Cost-Effectiveness of Linkage Case Management for Hospitalized People With HIV
Source: JAMA Netw Open. 2025 Nov 5;8(11):e2542105. doi: 10.1001/jamanetworkopen.2025.42105 (PMC12590294; doi:10.1001/jamanetworkopen.2025.42105)
Supplement: Supplement 1. — eAppendix 1. Microcosting analysis eAppendix 2. Disability-Adjusted Life-Years (DALYs) eAppendix 3. Statistical analyses eAppendix 4. Uncertainty eAppendix 5. Missing data eTable 1. Impact Inventory eTable 2. Unit Costs (2023 USD) eTable 3. Non-Medical and Other Services (NMOS) form eFigure. Sensitivity analyses eReferences. [file jamanetwopen-e2542105-s001.pdf]

Supplemental Online Content

Willkens M, Issarow B, Kisigo G, et al. Cost-effectiveness of linkage case management for hospitalized people with HIV. *JAMA Netw Open*. 2025;8(11):e2542105.
doi:10.1001/jamanetworkopen.2025.42105

eAppendix 1. Microcosting analysis

eAppendix 2. Disability-Adjusted Life-Years (DALYs)

eAppendix 3. Statistical analyses

eAppendix 4. Uncertainty

eAppendix 5. Missing data

eTable 1. Impact Inventory

eTable 2. Unit Costs (2023 USD)

eTable 3. Non-Medical and Other Services (NMOS) form

eFigure. Sensitivity analyses

eReferences.

This supplemental material has been provided by the authors to give readers additional information about their work.

© 2025 Willkens M et al. *JAMA Network Open*.

eAppendix 1 Microcosting analysis

A detailed microcosting analysis was conducted to estimate the real-world costs associated with the Daraja intervention, as described in Appendix 4 of Peck et al.[1–3] Intervention resource data were primarily collected via activity-based costing through site-visits accompanied by semi-structured interviews. Activity-based costing is a detailed approach that entails working closely with key study, clinical, and other personnel to determine which resources (e.g., labor, supplies) would be needed to implement and sustain the intervention in the "real-world". This process considered the reusability of study-generated resources, and the patient volume that could be achieved with these resources in a non-study context.

As per the prevailing economic-evaluation guidelines, cost-effectiveness analysis (CEA) incorporates the mean, per-participant cost of the intervention at a steady-state; thus, for the CEA we focused on the resources/costs associated with the sustainment phase of Daraja. Resources were categorized as "time-dependent" or "variable". Time-dependent resources are required throughout the intervention at predetermined intervals but remain constant irrespective of client volume. Variable resources are those that are required for each client served. The only time-dependent resource/cost was that of office space. Variable resources/costs included labor for face-to-face client sessions, check-in calls, patient referrals, locating patients, etc., and those associated with transportation.

Using the data discussed above from the microcosting analysis, a mean "real-world" cost was estimated for each potential point of contact associated with the Daraja intervention. Each observed participant contact was then weighted by the appropriate intervention unit cost, and the adjusted mean cost for Daraja was estimated using the methods described below in A.3.

eAppendix 2 Disability-Adjusted Life-Years (DALYs)

DALYs were calculated at the participant level by summing the years of life lost due to premature mortality (YLL) and the years lived with disability (YLD). Average life expectancy for men and women in Tanzania was obtained from the World Bank Group[4]; YLL was calculated as age at death subtracted from the average life expectancy for each participant who died prematurely. YLD represents a measure of time, weighted by the severity of an individual's disability. Disability weights, which quantify the severity of health loss associated with specific diseases or conditions and range from 0 (perfect health) to 1 (equivalent to death), were obtained from the 2021 Global Burden of Disease study, the most current year available.[5] Weights were assigned to all participants at each time point, according to the status of their HIV, anemia, and any other comorbid condition. Disability weights for participants with comorbid conditions were calculated according to the following equation:

$$DW_{combined} = (1\text{-}DW_1) - (1\text{-}DW_2).^{6}$$

YLD were calculated by estimating the area under the curve of the adjusted disability weight values (see A.3).

eAppendix 3 Statistical analyses

Predicted mean resource-cost and effectiveness measures were estimated using multivariable regressions within the generalized linear model (GLM) framework, and the statistical method of recycled predictions.[2] The GLM is a family of flexible statistical models that enable the mean and variance functions to be directly modeled according to the fit of the observed data. Recommended statistical tests support model selection, such as the modified Park test for the family, and the Pregibon link, Pearson correlation, and modified Hosmer-Lemeshow tests for selecting the link function.[2] The recycled predictions method involves calculating the adjusted-mean outcomes from a model by systematically varying specific independent variables of interest while keeping all other variables fixed at their observed values.

The following resource-cost categories were analyzed: Daraja, inpatient and outpatient care, CTC services, and other societal factors. Adjusted-mean disability weights and YLL were also estimated using the GLM framework, then incorporated into the formula described in A.2 to calculate DALYs. Regression models were defined a priori, according to established practices for studying individuals living with HIV, particularly those residing in Sub-Saharan Africa.[7–10] The following baseline participant measures were controlled for: gender, age, married or living

with someone, had previously used antiretrovirals, had completed of primary school (≥ 7 years), and enrollment-hospital fixed effects.

eAppendix 4 Uncertainty

The multivariable regressions used to estimate the adjusted mean cost and effectiveness measures were embedded within a nonparametric bootstrapping procedure to evaluate uncertainty around cost, effectiveness, and the incremental cost-effectiveness ratio (ICER).[2] The bootstrapping procedure generated 1,000 resampled datasets and adjusted mean cost and effectiveness values were estimated for each. These measures were used to obtain standard errors, which were then used within t-tests to assess differences between arms. Differences in adjusted-mean costs were independently tested for each resource category; subsequently, costs were summed according to perspective and differences in total costs were tested. All cost differences were analyzed over both the 3-month intervention, and the 12-month observation (intervention plus follow-up) periods. DALYs were also calculated for each of the resampled datasets, and differences tested at 3 and 12 months using the same methods deployed for the cost differentials.

Bootstrapped cost and effectiveness measures were also used to construct cost-effectiveness acceptability curves (CEACs) to evaluate the uncertainty around each ICER. Recall that cost-effectiveness is defined according to whether the ICER associated with the intervention is less than the decision-maker's willingness to pay for an additional unit of effectiveness. The CEAC displays the probability that an intervention is cost-effective across a range of potential stakeholder willingness-to-pay thresholds, according to the proportion of predicted mean ICERs that fall below those threshold values.[2]

eAppendix 5 Missing data

Missing data were monotonic and ranged from 0% at baseline to 3% (n=15) at 12 months for self-reported utilization of healthcare and societal resources. Moreover, missingness data did not differ significantly between arms across evaluation periods. Missing data bias was addressed within the GLM framework by employing inverse probability weighting (IPW). Both IPW and multiple imputation (MI) are well-established methods for effectively handling missing data; however, IPW may have advantages over MI in terms of efficiency and flexibility, as it eliminates the need for complicated and potentially unreliable imputation models within each bootstrap sample.[11,12]

© 2025 Willkens M et al. *JAMA Network Open*.

eTable 1: Impact Inventory

Sector	*Type of impact*	*Included in analysis from the following perspective?*		*Instrument*	*Notes on sources of evidence*
	(Categories impacted within each sector)	*TZ MOH*	*Societal*		
Health	*Health outcomes (effects)*				
	Disability-adjusted life-years, DALYs (patient)	X	X	Medical records; World Bank Group extraction; Disability weights	
	Medical costs				
	Paid for by third-party payers	X	X	NMOS	
	Paid for by providers	X	X	NMOS	
	Traditional healer services	-	X		
	Patient out-of-pocket	-	X		Data not available
	Future related medical costs	-	-		Outside time frame of study
	Future unrelated medical costs	-	-		Outside time frame of study
Informal healthcare sector					
Health	Patient time costs	NA	X	NMOS	
	Unpaid caregiver time costs	NA	-		Data not available
	Transportation costs	NA	-		Data not available
Non-healthcare sectors (with examples of possible items)					

© 2025 Willkens M et al. *JAMA Network Open*.

Sector	*Type of impact*	*Included in analysis from the following perspective?*		*Instrument*	*Notes on sources of evidence*
	(Categories impacted within each sector)	*TZ MOH*	*Societal*		
Productivity	Labor market earnings lost	NA	X	NMOS	
	Cost of unpaid lost productivity due to illness	NA	-		Data not available
	Cost of uncompensated household production	NA	-		Data not available
Consumption	Future consumption unrelated to health	NA	-		Outside time frame of study
Social services	Cost of social services as part of intervention	NA	-		Data not available
Criminal activity	Cost of crimes tangible + intangible	NA	-		Data not available
Education	Impact of intervention on educational achievement	NA	-		Data not relevant in the timeframe
Housing	Cost of intervention on home improvements (e.g., removing lead paint)	NA	NA		
Environment	E.g., production of toxic waste or pollution by intervention	NA	NA		
		Other Variables			
Demographics	All available	X	X	Study documents	Control variables in multivariable regressions
Previously used antiretrovirals		X	X	Study documents	Control variable in multivariable regressions

X = Included in analysis; - = not included in analysis; NA = not applicable; NMOS = Non-study Medical and Other Services Form

© 2025 Willkens M et al. *JAMA Network Open*.

eTable 2: Unit Costs (2023 USD)

Category	Cost (TZS)	Cost (USD)	Source
Inpatient, per day	25,000	10	Review of local medical payment schemes
Outpatient, per visit	20,000	8	Review of local medical payment schemes
Healer, per visit	70,000	28	Conversations with traditional healer attendees
CTC, per visit	20,000	8	Review of local medical payment schemes

© 2025 Willkens M et al. *JAMA Network Open*.

eTable 3: Non-Medical and Other Services (NMOS) form

<table>
<tr><th>QNo.</th><th>Code</th><th>Questions and Filters</th><th>Coding Categori</th></tr>
<tr><td>1201</td><td>COST1</td><td>Since your last assessment, how many nights did you stay in a hospital ward or clinic for your own health?</td><td>|__|__| nights</td></tr>
<tr><td>1202</td><td>COST2</td><td>How many visits did you make to an outpatient clinic (OPD) for your own health?</td><td>|__|__|</td></tr>
<tr><td>1203</td><td>COST3</td><td>How many visits did you make to a traditional healer for your own health?</td><td>|__|__|</td></tr>
<tr><td>1204</td><td>COST4</td><td>How many visits did you make to pharmacy or laboratory?</td><td>|__|__|</td></tr>
<tr><td>1205</td><td>COST5</td><td>How much have you spent out of your own pocket on healthcare (including hospital admissions, clinic visits, traditional healers, laboratory tests, and medications)?</td><td>Tsh. |__|__|__|__|__|_</td></tr>
<tr><td>1206</td><td>COST6</td><td>Did you earn any income through some kind of work or occupation in the past 3 months</td><td></td></tr>
<tr><td>1207</td><td>COST7M
COST7W
COST7D</td><td>On average, how much income do you earn?

If “Monthly” or “Weekly” skip to 1209</td><td>Tsh |__|__||__|__|__|__|__|__|
Tsh |__|__||__|__|__|__|__|__|
Tsh |__|__||__|__|__|__|__|_</td></tr>
<tr><td>1208</td><td>COST8</td><td>On average, how many days per week did you typically work</td><td>|__|__|days</td></tr>
<tr><td>1209</td><td>COST9</td><td>On average, how many hours have you spent on your healthcare per month (including travel and time in hospitals, clinics, with traditional healers, in labs and in pharmacies, etc.)?</td><td>|__|__|hours</td></tr>
<tr><td>1210</td><td>COST10</td><td>On average how many working hours did you miss per month due to health care?</td><td>|__|__|hours</td></tr>
</table>

© 2025 Willkens M et al. *JAMA Network Open*.

eFigure. Sensitivity analyses

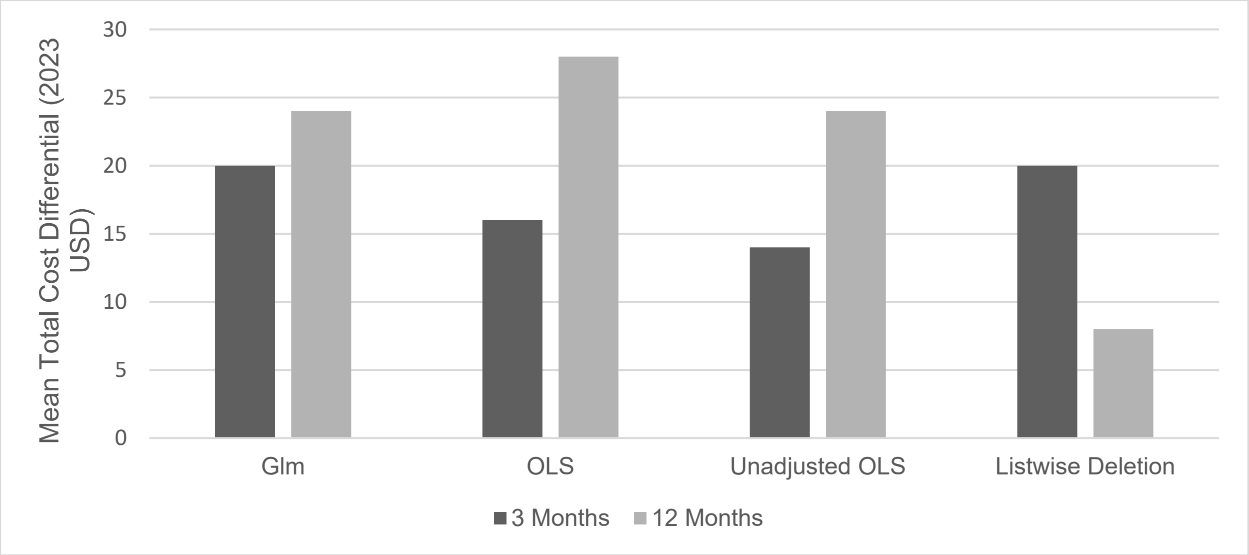

© 2025 Willkens M et al. *JAMA Network Open*.

eReferences

1. Peck RN, Issarow B, Kisigo GA, Kabakama S, Okello E, Rutachunzibwa T, et al. Linkage Case Management and Posthospitalization Outcomes in People With HIV. JAMA. 2024 Mar 26;331(12):1025.

2. Glick H, Doshi J, Sonnad S. Economic evaluation in clinical trial. Polsky D, editor. New York: Oxford University Press; 2014.

3. Neumann P, Sanders G, Russell L, Siegel J, Ganiats T. Cost-effectiveness in health and medicine. . 2nd ed. Neumann P, Sanders G, Russell L, Siegel J, Ganiats T, editors. New York: Oxford University Press; 2017.

4. World Bank Group. Life expectancy at birth, total (years). [cited 2024 Nov 18]; Available from: data.worldbank.org

5. Institute for Health Metrics and Evaluation. Global Burden of Disease Study 2021 (GBD 2021) Disability Weights. [cited 2024 Jun 11]; Available from: www.ghdx.healthdata.org

6. Hilderink HBM, Plasmans MHD, Snijders BEP, Boshuizen HC, Poos MJJC, van Gool CH. Accounting for multimorbidity can affect the estimation of the Burden of Disease: a comparison of approaches. Archives of Public Health. 2016 Dec 22;74(1):37.

7. Toska E, Zhou S, Laurenzi CA, Haghighat R, Saal W, Gulaid L, et al. Predictors of secondary HIV transmission risk in a cohort of adolescents living with HIV in South Africa. AIDS. 2022 Feb 1;36(2):267–76.

8. Wamoyi J, Stobeanau K, Bobrova N, Abramsky T, Watts C. Transactional sex and risk for HIV infection in sub-Saharan Africa: a systematic review and meta-analysis. J Int AIDS Soc. 2016 Jan 2;19(1).

9. Hudelson C, Cluver L. Factors associated with adherence to antiretroviral therapy among adolescents living with HIV/AIDS in low- and middle-income countries: a systematic review. AIDS Care. 2015 Jul 3;27(7):805–16.

10. Frijters EM, Hermans LE, Wensing AMJ, Devillé WLJM, Tempelman HA, De Wit JBF. Risk factors for loss to follow-up from antiretroviral therapy programmes in low-income and middle-income countries. AIDS. 2020 Jul 15;34(9):1261–88.

11. Jalali A, Tamimi RM, McPherson SM, Murphy SM. Econometric Issues in Prospective Economic Evaluations Alongside Clinical Trials: Combining the Nonparametric Bootstrap With Methods That Address Missing Data. Epidemiol Rev. 2022 Dec 21;44(1):67–77.

12. Seaman SR, White IR. Review of inverse probability weighting for dealing with missing data. Stat Methods Med Res. 2013 Jun 10;22(3):278–95.

13. Husereau D, Drummond M, Augustovski F, de Bekker-Grob E, Briggs AH, Carswell C, et al. Consolidated Health Economic Evaluation Reporting Standards 2022 (CHEERS 2022) statement: updated reporting guidance for health economic evaluations. BMC Med. 2022 Jan 12;20(1):23.

© 2025 Willkens M et al. *JAMA Network Open*.
