# Supplementary material for: Cost-Effectiveness of Linkage Case Management for Hospitalized People With HIV
Source: JAMA Netw Open. 2025 Nov 5;8(11):e2542105. doi: 10.1001/jamanetworkopen.2025.42105 (PMC12590294; doi:10.1001/jamanetworkopen.2025.42105)
Supplement: Supplement 2. — Data Sharing Statement [file jamanetwopen-e2542105-s002.pdf]

Data Sharing Statement

Willkens. Cost-Effectiveness of Linkage Case Management for Hospitalized People With HIV. *JAMA Netw Open*. Published November 05, 2025. doi:10.1001/jamanetworkopen.2025.42105

Data
Data available: Yes
Data types: Deidentified participant data
How to access data: Contact for data-sharing agreement: rnp2002@med.cornell.edu.
When available: With publication

Supporting Documents
Document types: Statistical/analytic code, Informed consent form
How to access documents: Contact for data-sharing agreement: rnp2002@med.cornell.edu.
When available: With publication

Additional Information
Who can access the data: Who can access the data: According to our agreement with the study sponsor and the Institutional Review Boards (IRBs) who approved this protocol, the final dataset will include deidentified data on demographic characteristics, medical history, psychosocial measures, survival, HIV clinic linkage and retention, ART adherence, and HIV viral loads. Even though the final dataset will be stripped of identifiers prior to release for sharing, we believe that there remains the possibility of deductive disclosure of subjects with unusual characteristics. Therefore, we will make the dataset available to users under a data-sharing agreement that includes the following: a commitment to use the data for research purposes and not participant identification, commitment to securing the data, and a commitment to destroying the data after analyses are completed. A data dictionary and analytic code will be provided with any shared data.
Types of analyses: Research purposes only.
Mechanisms of data availability: Signed data access agreement.
